# The terminal period findings of late-diagnosed fibrodysplasia ossificans progressiva

**DOI:** 10.3205/000326

**Published:** 2023-07-11

**Authors:** Emrah Doğan, Hüseyin Aydoğmuş, Cenk Elibol, Sinem Aydoğmuş

**Affiliations:** 1 Department of Radiology, Muğla Sıtkı Koçman University, Muğla, Turkey; 2 Department of Physical Medicine and Rehabilitation, Muğla Sıtkı Koçman University Education and Research Hospital, Muğla, Turkey; 3 Department of Radiology, Muğla Sıtkı Koçman University Education and Research Hospital, Muğla, Turkey

## Abstract

Fibrodysplasia ossificans progressiva (FOP) is an autosomal dominant rare disease characterized by foot deformities and concomitant heterotopic ossifications. Theoretically, in the absence of early diagnosis and medication, the patient’s outcome will be poor. The patients are usually diagnosed at an early age. Hence, encountering a non-treated and terminal-period patient is rare. Our case was unique because it showed the clinical picture and atypical radiological distribution of a 20-year-old, terminally ill untreated female patient. She had hallux valgus, heterotopic ossifications and multiple osteochondromas that were detected in the right clavicula, the posterior arch of the 9^th^ rib, the bilateral tibia and fibula. Atypically, heterotopic ossifications were not present in the soft tissues of the neck. Hand deformity, cardiac anomaly, or mental retardation was not observed. It was a sporadic case. The presentation with neurological symptoms was also atypical.

## Abstract (de)

Fibrodysplasia ossificans progressiva (FOP) ist eine autosomal-dominante, seltene Krankheit, die sich durch Fußdeformitäten und gleichzeitige heterotope Verknöcherungen auszeichnet. Wird die Krankheit nicht frühzeitig diagnostiziert und medikamentös behandelt, sind die Aussichten für die Patienten theoretisch schlecht. Die Diagnose erfolgt in der Regel in einem frühen Alter der Patienten. Daher ist es selten, einen unbehandelten Patienten im Endstadium anzutreffen. Unser Fall war einzigartig, weil er das klinische Bild und die atypische radiologische Verteilung bei einer 20-jährigen, unheilbar kranken, unbehandelten Patientin zeigte. Sie hatte einen Hallux valgus, heterotope Verknöcherungen und multiple Osteochondrome, die in der rechten Clavicula, dem hinteren Bogen der 9. Rippe und beidseitig in Tibia und Fibula entdeckt wurden. Heterotopische Verknöcherungen in den Weichteilen des Halses waren untypischerweise nicht vorhanden. Handdeformitäten, Herzanomalien oder geistige Entwicklungsstörungen wurden nicht beobachtet. Es handelte sich um einen sporadischen Fall. Das Auftreten neurologischer Symptome war ebenfalls atypisch.

## Introduction

FOP is a rare disease with an incidence of 1/2,000,000 [1]. Most of the patients are sporadic cases resulting from new mutations, although FOP is an autosomal dominant disease [2]. The disabilities resulting from progressive heterotopic calcifications and congenital big toe anomalies (especially hallux valgus) constitute the main picture of FOP. Osteochondromas and atypical congenital malformations are the most common accompanying findings [3]. Infection, immunization, trauma and surgical procedures can trigger heterotopic calcifications [4]. There is no known effective treatment method for FOP so far [3]. However, early diagnosis is essential for decelerating progression, passing the acute flare-ups more easily and preventing unnecessary operative interventions [5]. Our case was a late-diagnosed patient who did not take palliative or curative treatment. We present the case of a 20-year-old female patient accompanied by the clinical and radiological findings.

## Case description

A 20-year-old female patient was admitted to the hospital with restrictions of joint movement, back and neck pain, and difficulty in walking. In the history of the patient, she was considered a normal newborn since there was no developmental anomaly. The initial problems started with swelling in the back at four years old. At the end of the first decade, complaints of severe pain, joint stiffness, and swelling in the back and extremities became obvious. The patient visited many hospitals without a diagnosis being made. However, she did not take any treatment except analgesics (NSAIDs, acetaminophen, paracetamol) and various palliative drugs (morphine, midazolam), especially during exacerbation and pain attacks. FOP was first suspected in 2015, taking into account hallux valgus and atypical ossifications. The genetic test performed in an external medical center had confirmed the FOP diagnosis.

The patient had a history of epileptic seizures that started in 2016. The first attack started with the blinking of both eyes, locking in the teeth, loss of consciousness, and spasm in the arms. Except for the bite mark on the patient’s tongue, the first post-seizure neurological examination was completely normal. No acute pathology was observed in the cranial BT. Tegretol at a dose of 200 mg twice a day has been prescribed. Until 2018, when the patient was admitted to the emergency department with recurrent epileptic seizures, no attack was noted for a long time. From the anamnesis, it was understood that the patient had been hospitalized in the general surgery ward due to abdominal muscle rupture two days before the seizure and had not taken epilepsy drugs for this reason. Thus, recurrence of seizures was attributed to the lack of medication.

Emergency treatment consisted of the infusion of 5 ampoules of phenytoin. Brain CT performed at this time was normal. At the patient’s discharge, Tegretol 200 mg was increased to one and a half tablets twice a day. Despite restarting the medication, the patient had 3 attacks between January and April 2018.

In October 2018, subarachnoid and interhemispheric hemorrhage occurred as a result of the trauma related to fainting and falling. The hemorrhage spontaneously resorbed.

In the obstetrical and gynecological history of the patient, recurrent abortions at five and two months in 2016 and 2018 were reported. After abortions, the patient was followed up for secondary amenorrhea. Her hormone profile was normal. Uterus didelphis, right uterine horn stenosis, fundal myoma uteri (size 51x47 mm) and a left ovarian follicle cyst (size 25 mm) were the detected gynecological pathologies in ultrasound and hysterosalpingography. Secondary amenorrhea was attributed to multiple abortuses, anatomical pathologies and epileptical drug.

The patient presented to the physical therapy outpatient clinic several times in January 2016, April 2017, January 2018, and December 2019 because of severe pain. Stretching, opening, and closing exercises were recommended. The physiotherapy program was performed with the continuous passive motion (CPM) machine.

**Figure 1:**
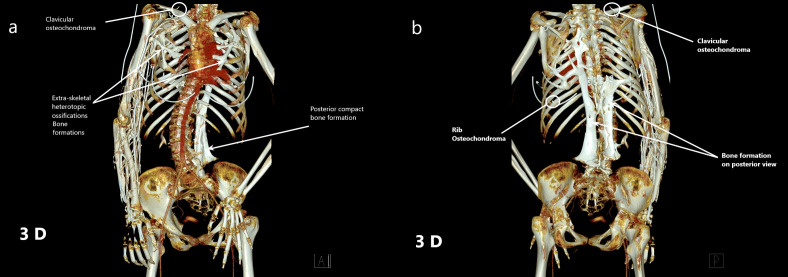
3D reconstruction images; the extra-skeletal bone formations and heterotopic ossifications around the chest, clavicular osteochondroma in the middle part of right clavicula, compact bone formations along with the vertebral axis

**Figure 2:**
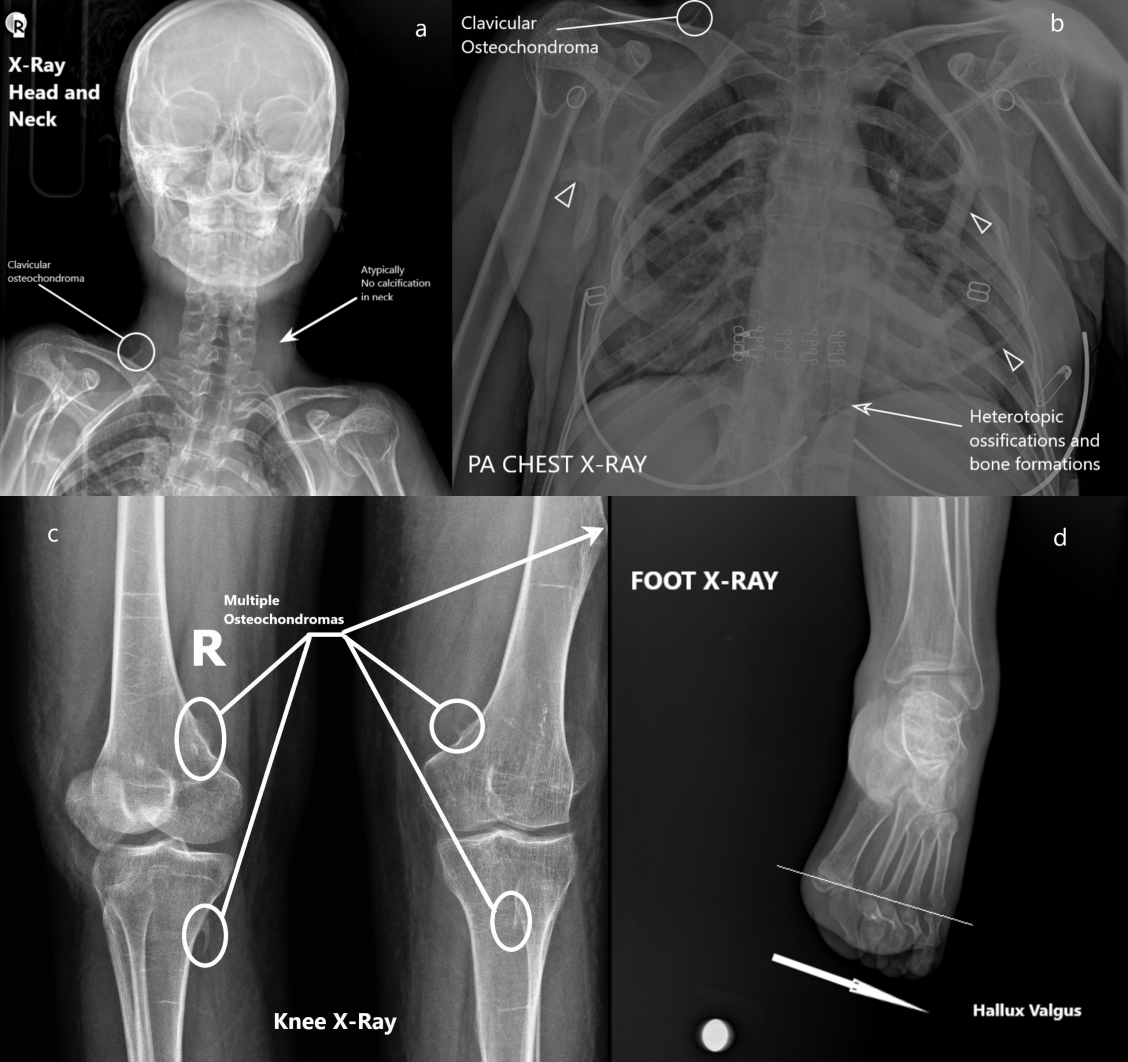
X-ray graphs; a) atypically no calcifications or ossifications in the neck, right clavicular osteochondroma; b) heterotopic ossifications and bone formations around the chest (arrowhead), compact bone formations along with vertebral axis, clavicular osteochondroma; c) multiple osteochondromas located around the knee joint; d) hallux valgus anomal

In the pulmonary and cardiac examination, respiratory sounds were bilaterally normal. No rales, rhonchi or pathological cardiac sounds (S1 + S2 + additional sound or murmur) were detected in the auscultation. The abdominal examination was also normal. No abdominal sensitivity, rebounds or defense was found. In the evaluation of the skeletal system from up to down, there was flattening in the neck. The shoulders’ abduction and left knee movement were limited. Difficulty in the extension of the waist and feet was present. Bilateral Babinski reflex was negative. Bilateral edema was present. Venous Doppler USG was requested. There was no vascular pathology. According to radiological algorithm, X-rays were taken first. Following the discovery of abnormalities, chest CT scans were performed. At the result of these evaluations, multiple linear heterotrophic bone formations that cover the chest were seen (paraspinal muscle calcifications). There were bridges between ectopic ossifications (Figure 1 (Fig. 1)). Scoliosis, ankylosis in bony structures and thickening of the paravertebral ligaments were present. Unlike the expected course of the disease, heterotopic neck ossifications were not detected (Figure 2a (Fig. 2)). Multiple osteochondromas in the right clavicle (Figure 1 (Fig. 1), Figure 2a (Fig. 2)), the posterior arch of the 9^th^ rib (Figure 1 (Fig. 1)) with bridge ossifications (Figure 2b (Fig. 2)), metaphysis location of the right femur, distal diaphysis of the left femur, proximal metaphysis of the right femur, and fibula head (Figure 2c (Fig. 2)) were seen. Hallux valgus anomaly was present in both feet (Figure 2d (Fig. 2)). Table 1 (Tab. 1) shows common, ancillary and uncommon findings.

**Figure 3:**
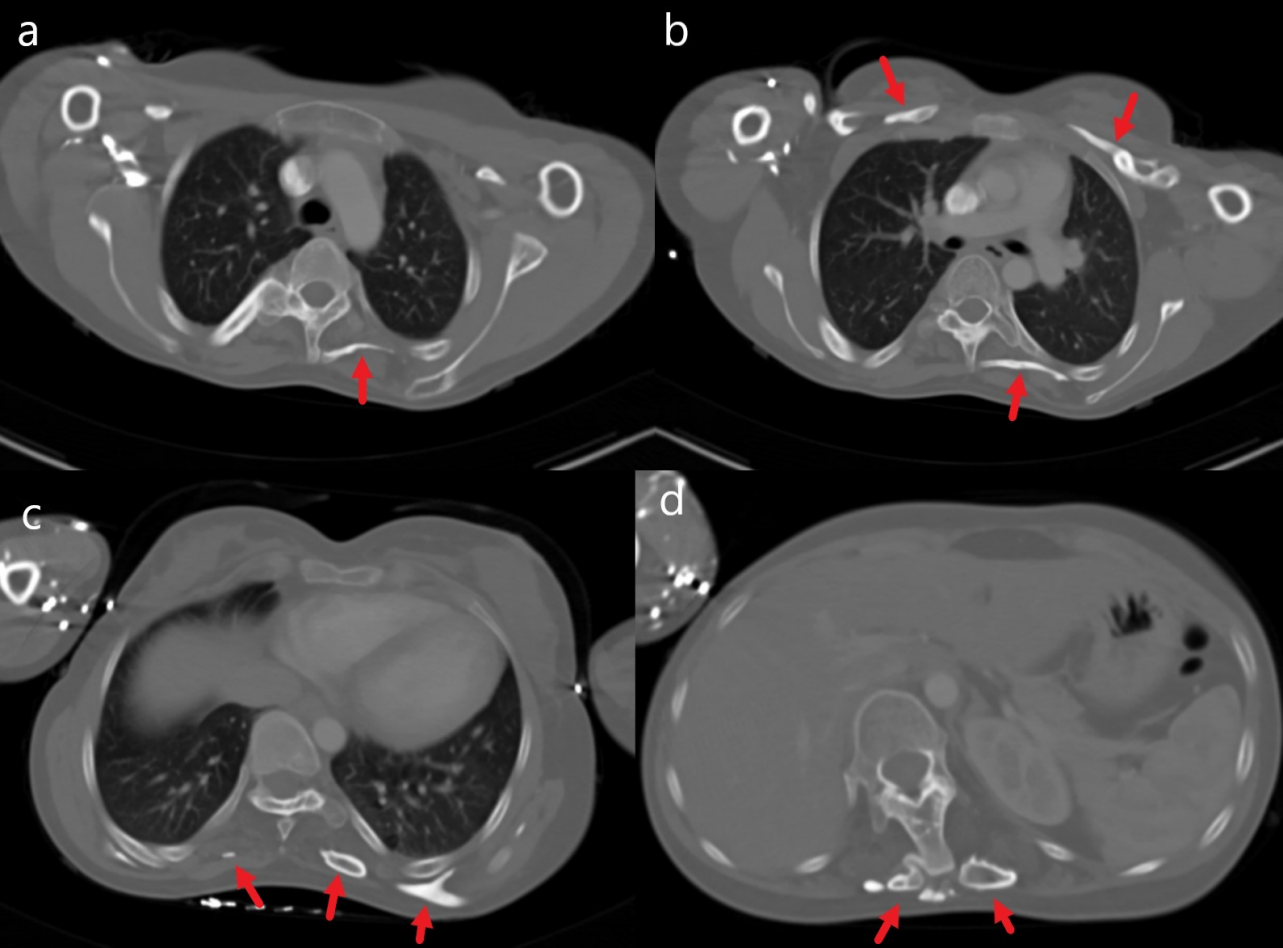
Axial CT images show heterotopic ossifications in the paravertebral and the perithoracic areas; a) upper thoracic level; b) subcarinal level; c) lower thoracic level; d) upper abdominal level

CT images of the axial section showed that the heterotopic ossifications overlapped with the perithoracic muscle lines. The subcutaneous regions were intact (Figure 3 (Fig. 3)).

The only treatment recommended to the patient was follow-up and steroids at the time of the flare-ups. The patient consent form was signed on 18 April 2020 by the patient herself.

## Discussion

FOP is a rare genetic disease characterized by progressive ectopic pathologic bone formations in skeletal muscles resulting from trauma or infection [6]. It is also called ‘Munchmeyer’s disease’ or ‘myositis ossificans progressive’ [7], [8]. Congenital foot anomalies are characteristic [9]. John Freke published the first scientific paper about FOP in 1740. However, FOP was probably first described long before, by Guy Patin [10]. The first name of this disease was ‘Stoneman’s disease’.

The number of FOP patients in the world is limited. There are currently 800 known patients [10]. Although calculating the presence of 2,000 alive cases in consideration of the incidence rate, the world population, and the patient’s lifespan, more than half of the patients are not present in medical records.

It is difficult to estimate a precise age, but symptoms seem to begin at around five years of age [11]. The genetic background of FOP is related to the overexpression of bone morphogenic protein (BMP 4). The mutation affecting the type 1 receptors of this protein activates osteogenesis. The responsible mutational gene from osteogenesis is the ACVR1 (activin A receptor), located in the second chromosome [12].

At first, heterotopic ossifications start from the neck and progress to the thoracic and lumbar regions. In other words, FOP has a cranio-caudal evolution [13]. The sternocleidomastoid muscle is considered the initiation point of ossifications. The muscle groups of the ocular region, tongue, larynx, heart, diaphragm, abdominal wall, and smooth muscles are not involved [13]. Early diagnosis is essential for avoiding unnecessary surgical intervention, overcoming acute flare-up periods with fewer complications, patient comfort, and slowing down progression [5]. Our patient was diagnosed 5 years ago at the age of 15 in the terminal period. She had widespread heterotopic ossifications and bridges. Severe contractures were observed. However, heterotopic ossifications were not found in the neck. Thus, the appearance was atypical for FOP.

X-ray images are technically enough for bone and ossification assessment. They can show bone bridges, various skeletal deformities, and the ectopic ossification of soft tissues. Also, hand and foot deformities can easily be evaluated with X-ray imaging [14]. Advanced radiological imaging can be necessary for more detailed evaluation. CT scans can show ossifications with precision. Magnetic resonance imaging (MRI) is useful to show early-stage lesions, especially before ossifications [15]. The laboratory values are generally normal [9]. FOP is diagnosed with clinical and radiological findings. The biopsy is contraindicated because it triggers ossifications [16].

The general clinical picture is the combination of congenital anomalies of feet and progressive ossifications. Congenital skeletal anomalies characteristically affect the big toe in all of the cases. These anomalies are deviation (hallux valgus) and rigidity [1]. Hallux valgus is the indispensable (sine qua non) finding of FOP. Sharp first metatarsal bone and first finger shortness can be present. Besides, some hand deformities associated with FOP can also be found. Common hand deformities are short first metacarpal and clinodactyly in the 5^th^ finger. The cervical vertebra malformation, short wide femoral neck, cardiac conduction defects, deafness, baldness, and moderate mental retardation are other reported anomalies in FOP [17]. Osteochondromas are seen in 50% of the cases [10]. Indeed, our patient had multiple osteochondromas in the bilateral leg, ribs, and clavicula.

There are not many diseases in the differential diagnosis with FOP, since the main picture of the disease is clear with foot anomalies and accompanying ossifications. Myositis ossificans as well as endocrinological genetic and non-genetic dystrophies can be considered in the differential diagnosis.

Our patient had multiple osteochondromas. If other findings were not present, hereditary multiple exostoses may be considered [18]. Probably, the only finding that could be found at birth is hallux valgus, and in this case, FOP should come to mind as a diagnosis. Thus, the genetic test should be recommended to patients with hallux valgus [19].

Kitterman reported that 51% of patients with FOP suffered from at least one neurological symptom.

The rate of neurological symptoms in females was higher than in males. Recurrent headache and sensory anomalies include a large proportion of neurological findings detected. According to Kitterman, the finding of recurrent seizures seen in our patient is one of the atypical neurological findings, with an incidence of 2.8% [20].

Our patient had secondary amenorrhea due to an unexplained reason. However, in our case it could be related to other causes, since Elsaiti et al. highlighted that secondary amenorrhea was extremely rare compared to primary amenorrhea in FOP [21].

Concerning treatment, although steroid and non-steroidal anti-inflammatory drugs are used during acute flare-up periods, there is no evidence of their effectiveness. Besides, steroids have side effects at a high rate. Theoretically, amino bisphosphonates, inhibitors of leukotrienes, and stabilizers of mastocytes are other potential medical instruments preventing inflammatory edema, fibroproliferation, and angiogenesis. Dorsomorphin (BMC type 1 receptor inhibitor) and palovarotene are new generation agents [13]. Furthermore, radiation therapy has also been tried for heterotopic ossifications [22]. Genetic studies on the disease are ongoing. However, a satisfactory result has not been obtained yet.

According to the literature, the cause of death is usually complications resulting from vertebral deformations such as respiratory failure, pneumonia, atelectasis, or right cardiac failure [1], [7]. Life expectancy is around the fourth decade [5].

## Conclusion

Patients with FOP are generally diagnosed at an early age. Theoretically, in the absence of early diagnosis and medication, the patient’s evolution will be detrimental. Therefore, encountering a non-treated and terminal-period patient is very rare. Our case was unique because it showed the clinical picture and unusual radiological distribution of a 20-year-old, terminally ill untreated female patient.

It was a sporadic case with no family history. The radiological findings have great importance for FOP diagnosis. On the one hand, the absence of ossifications in the neck is very atypical for FOP, and on the other hand, the presence of multiple osteochondromas and diffuse heterotopic ossifications were quite remarkable in our patient.

## Notes

### Acknowledgments

We would like to thank Marwa Mouline Dogan for her substantial support.

### Competing interests

The authors declare that they have no competing interests.

## Figures and Tables

**Table 1:**
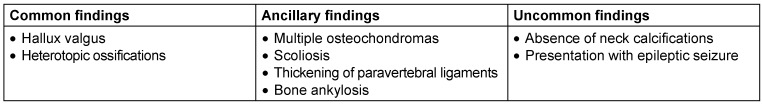
Common, ancillary and uncommon findings
